# Statistical or biological significance?

**DOI:** 10.1186/s12915-015-0198-1

**Published:** 2015-11-05

**Authors:** Emma Saxon

**Affiliations:** BMC Biology, BioMed Central, 236 Gray’s Inn Road, London, WC1X 8HB UK

## Abstract

Oat plants grown at an agricultural research facility produce higher yields in Field 1 than in Field 2, under well fertilised conditions and with similar weather exposure; all oat plants in both fields are healthy and show no sign of disease. In this study, the authors hypothesised that the soil microbial community might be different in each field, and these differences might explain the difference in oat plant growth. They carried out a metagenomic analysis of the 16 s ribosomal ‘signature’ sequences from bacteria in 50 randomly located soil samples in each field to determine the composition of the bacterial community. The study identified >1000 species, most of which were present in both fields. The authors identified two plant growth-promoting species that were significantly reduced in soil from Field 2 (Student’s t-test *P* < 0.05), and concluded that these species might have contributed to reduced yield.

## Comment

**Fig. 1:**
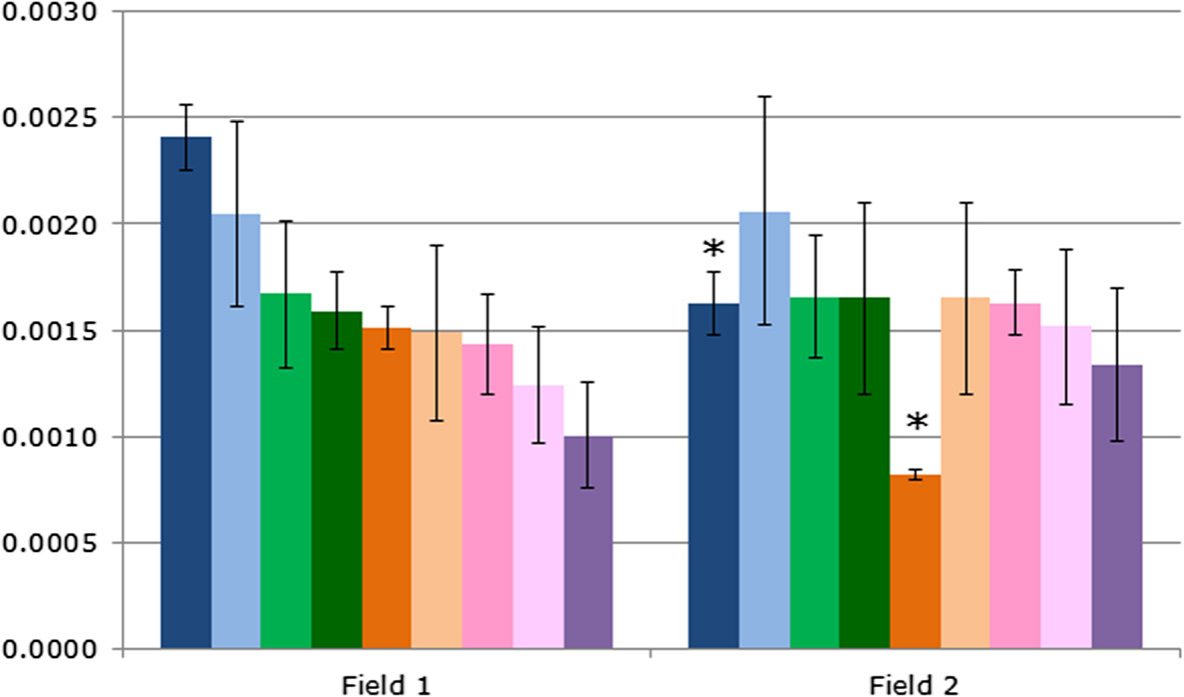
The proportion of nine known plant growth-promoting bacterial species detected in the soil bacterial community of two fields. Oat plant yield was greater in Field 1 than Field 2; two of the growth-promoting bacterial species were found at a significantly lower level in Field 2 than Field 1 (Student’s t-test *P < 0.05; error bars show standard deviation)

The previous example in this series addressed the problem of correcting for multiple comparisons. But even if the authors’ findings were significant after applying a correction, there is still another issue: the authors determined the levels of each bacterial species as a percentage of the whole community, and not as their number per unit of soil, which is more relevant to the potential biological effect of any difference between the fields. Each sample sent for sequencing was taken from 1 g soil and contained ~500,000 sequences, each assumed to correspond to one bacterial cell: the two species where the difference between the two soils was significant differ by only 0.0007 and 0.0008 %, corresponding to just 350–400 cells [Fig. 1]. This small number of bacterial cells is unlikely to have had a significant effect on oat plant growth; although statistically significant, the results are not likely to be biologically significant.

Another potential problem with this study is that although the 16 s ribosomal sequence is commonly used to identify bacterial species in metagenomic studies, many species have more than one copy of the 16 s sequence in their genome. Studies of bacterial abundance, such as this one, may, therefore, overestimate the number of bacterial species with a 16 s copy number greater than one. In a 2012 study, Kembel and coworkers [1] illustrated the importance of this problem by applying estimations of copy number to previously published metagenomic data sets, based on known copy numbers from diverse bacterial species. This adjustment for 16 s copy number changed some of the original outcomes reported in the published studies: in an oceanic data set, the ninth most abundant taxon became the second most abundant, and in a human microbiome study, the bacterial community found in the ear became more similar to that in the nostril rather than the sole of the foot — a more intuitive result.

The authors of that study created software designed to account for copy number, which can be used in conjunction with the open-source software already used for analysing metagenomic data sets, such as QIIME (Quantitative Insights Into Microbial Ecology) [1]. Correcting for copy number can also be carried out using PICRUSt (Phylogenetic Investigation of Communities by Reconstruction of Unobserved States) [2].
